# Cerebral Salt Wasting Syndrome in the Setting of Hypovolemic Isotonic Hyponatremia: A Case Report

**DOI:** 10.7759/cureus.49928

**Published:** 2023-12-04

**Authors:** Gerik Cervantes, Joshua Amaya, Pardis A Zarandi, Diana Wilson, Sandeep K Shori

**Affiliations:** 1 Medicine, Anne Burnett School of Medicine, Texas Christian University, Fort Worth, USA; 2 Internal Medicine, Baylor Scott and White All Saints Medical Center, Fort Worth, USA; 3 Neurosurgery, Baylor Scott and White All Saints Medical Center, Fort Worth, USA; 4 Graduate Medical Education, Baylor Scott and White All Saints Medical Center, Fort Worth, USA

**Keywords:** cerebral salt wasting syndrome, multifactoral hyponatremia, euvolemic hyponatremia, treatment guidelines for hyponatremia, differential diagnosis of hyponatremia, hypovolemia, isotonic hyponatremia, hypotonic hyponatremia, acute hyponatremia

## Abstract

Cerebral salt wasting syndrome (CSWS) is a condition characterized as the loss of sodium secondarily due to an intracranial process, commonly following the neurosurgical resection of mass lesions. This condition leads to a hypovolemic hypotonic hyponatremia. The identification of this syndrome is often mistaken for syndrome of inappropriate anti-diuretic hormone (SIADH). The treatment for both conditions is vastly different. Given the risk of mortality when balancing sensitivities in hyponatremia and its commonality in clinical scenarios, a distinction is crucial. In this case report, we discuss a patient who presented with CSWS following the surgical resection of a pituitary adenoma. She subsequently developed hypernatremia, treated with DDAVP for the suspicion of diabetes insipidus. Once this was discontinued, she further presented with worsening hyponatremia. This hyponatremia persisted even after the discontinuation of DDAVP, with no significant intervention leading to hypovolemic isotonic hyponatremia, supporting a diagnosis of CSWS. Our findings stress the importance of the proper identification of hyponatremia with guided treatment following neurosurgical intervention and give physicians an insight into the anomalies of hyponatremia that should be further discussed.

## Introduction

Hyponatremia, defined as a serum sodium concentration of <135 mmol/L, is the most common electrolyte problem in medicine worldwide. It often accompanies pathologies involving the central nervous system. These conditions include intracranial hemorrhage (most commonly subarachnoid hemorrhage), traumatic brain injury (TBI), stroke, and tumors, which can cause clinical scenarios surrounding low serum sodium levels [1,2]. First described by Peters et al. in 1950, cerebral salt wasting syndrome (CSWS) was described as natriuresis, hyponatremia, and hypovolemic dehydration in patients with intracranial pathologies [3]. The challenge lies in differentiating this clinical entity from syndrome of inappropriate anti-diuretic hormone (SIADH), which presents similarly. Both conditions account for the most common cause of hyponatremia in hospitalized patients, but this incidence increases to 50% in neurological patients [4].

Following this distinction in syndromes of hyponatremia, diagnosis can be challenging due to its various similarities. SIADH, characterized by elevated levels of anti-diuretic hormone (ADH), causes water retention by increasing the permeability of water in the collecting duct of the kidneys, reducing urinary output and ultimately resulting in euvolemic hypotonic hyponatremia. Similarly, hyponatremia in CSWS is characterized as the renal loss of sodium secondarily due to intracranial processes including the increase of brain natriuretic peptide, which inhibits renal sodium transport [5]. CSWS is further classified as hypovolemic hypotonic hyponatremia. Although similar, the differentiation of these two disorders focuses on volume status, though this has been challenged [6]. Nevertheless, hyponatremia, if left untreated, can result in seizures, worsening cerebral edema, and death. Given the commonality of hyponatremia in clinical settings, the distinction between these two conditions is crucial, as treatment parameters are dramatically different.

In this case report, we discuss a patient admitted to the intensive care unit (ICU) following the transsphenoidal resection of a pituitary adenoma who presented with hyponatremia six days postoperatively during her stay in the ICU. This led to a unique and incredibly rare case of CSWS characterized clinically as hypovolemic isotonic hyponatremia.

## Case presentation

**Figure 1:**
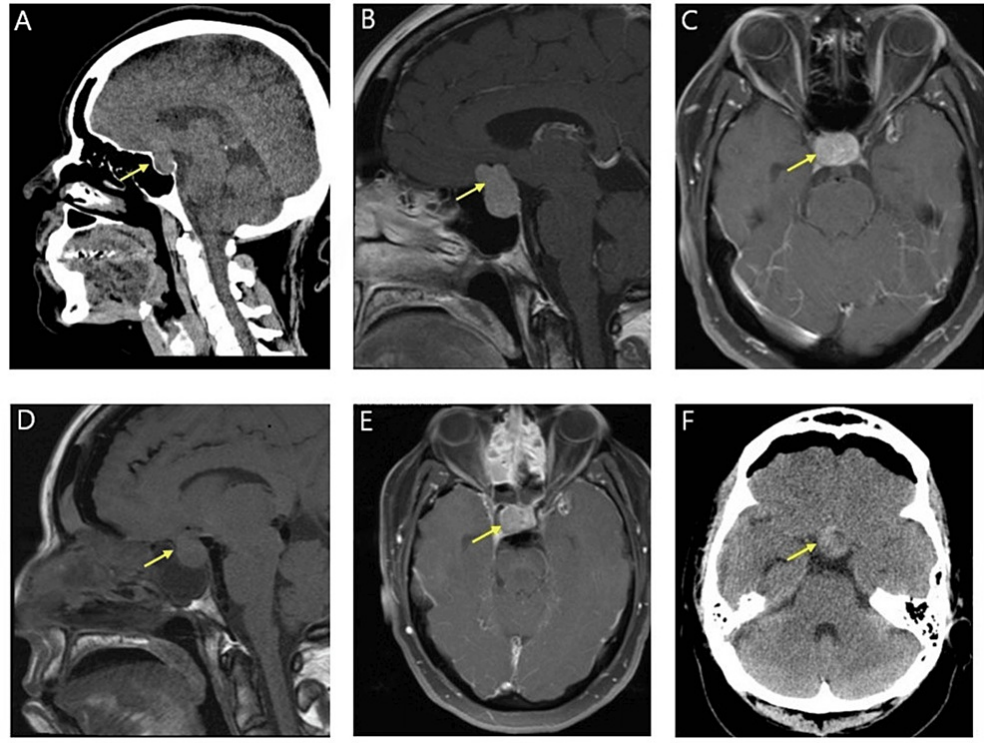
Demonstration of pituitary macroadenoma on CT and MRI CT of the sinus with contrast (sagittal view) (A) and sagittal T1 (B) and axial (C) MRI of the brain with contrast reveal a pituitary sellar/suprasellar 1.8 × 2.5 × 1.9 cm mass, consistent with pituitary macroadenoma prior to resection. Post transsphenoidal resection, residual adenoma is demonstrated within the sella and suprasellar region on sagittal (D) and axial (E) T1-weighted MRI of the brain with contrast. Subsequently, the patient underwent repeat transsphenoidal resection to remove the residual tumor and repair the CSF leak. A follow-up CT of the brain without contrast (axial view) (F) exhibits hyperdense material within the sella representing postoperative blood products versus residual tumor CT, computed tomography; MRI, magnetic resonance imaging; CSF, cerebrospinal fluid

Our patient is a 46-year-old female who presented to the emergency department with a chief complaint of left-sided tingling and numbness in her face and fingers. She noted similar symptoms three years prior but now presented with associated lightheadedness. Her past medical history includes hypertension, gastroesophageal reflux disease, and anxiety, and she does not take any medication daily. Her social history consists of daily alcohol intake and smoking half a pack a day. After the suspicion of an acute intracranial process was considered, non-contrast computed tomography (CT) was completed to rule out the potential for a stroke, as well as electrocardiogram and laboratory metabolic and blood count panels. Initial CT demonstrated a 2.1 cm suprasellar mass lesion with no evidence of ischemia or other processes. On further evaluation, magnetic resonance imaging (MRI) demonstrated a 1.8 × 2.2 × 2.8 cm pituitary macroadenoma with the compression of the optic chiasm and the pre-chiasmatic optic nerves (Figure 1A, 1B, 1C). She was managed appropriately, and a surgery date was determined.

She underwent transsphenoidal endoscopic and microscopic surgical resection of pituitary macroadenoma and was admitted to the ICU for observation. The following day, postoperative day 1, she was notably urinating large volumes of fluid, out of proportion to her fluid intake, with the intake being approximately 4 L and output approximately greater than 8 L within 24 hours postoperatively. She stated that she felt thirsty and overall had signs of volume depletion on physical examination including dry mucous membranes and decreased skin turgor. Laboratory work demonstrated a hypernatremic state; laboratory values are listed in Table 1. This was presumably central diabetes insipidus, and the patient was started on desmopressin 1 mcg subcutaneously (DDAVP). Her vitals were stable postoperatively, and she was continuously observed.

**Table 1:** Laboratory values from the patient’s postoperative first day to discharge, with laboratory results following the initiation of normal saline *Normalization of sodium following normal saline (0.9%) FENa: fractional excretion of sodium

	Postoperative Day 1	Postoperative Day 6	Postoperative Day 7	Postoperative Day 8	Post Initiation of Normal Saline	Post Residual Resection Day 1	Discharge	Reference
Serum Sodium	148	130	129	130	136*	145	140	136-145 mEq/L
Serum Osmolality	-	278	-	283	-	299	-	275-285 mOsm/kg
Urine Osmolality	-	422	-	577	-	146	-	<100 mOsm/kg
Urine Sodium	-	142	-	111	-	23	-	>20 mmol/L
FENa	-	2.8%	-	-	-	-	-	<1%

Her sodium normalized one day after surgery, and her polyuria improved. Of note on postoperative day 3, she had a cerebrospinal fluid (CSF) leak that required surgical repair, which was uneventful. On postoperative day 6, her sodium levels began down trending, DDAVP was discontinued, and nephrology was consulted. Serum sodium levels began down trending further. This surprised the critical care team and neurosurgery as her serum sodium was initially hypernatremic and transitioned into declining sodium levels. As her hyponatremia worsened without significant intervention, further evaluation of the patient’s fluid status was completed. Her serum osmolality at this state was within normal limits, and urine osmolality, urinary sodium, and fractional excretion of sodium were elevated as shown in Table 1. The patient was euvolemic on physical examination, with moist mucous membranes, normal capillary refill, and skin turgor, yet she reported poor oral intake. Her pulse pressure at this time was within normal limits at 44. Given her laboratory values, she demonstrated a hypotonic euvolemic hyponatremic state and clinically appeared to reflect iatrogenic SIADH. Her sodium levels began to decline, ultimately reaching a peak decline of 129 mEq/L on postoperative day 7. Her serum osmolality was reassessed on postoperative day 8, with a mild increase but still within normal parameters. Her urinary osmolality was increasing with a slight decrease in urine sodium, which remained elevated, classifying her hyponatremia as isotonic. It is important to note that the patient was discontinued on desmopressin 24 hours prior, given the 2.7-4.6 hour half-life of DDAVP. Laboratory values did not demonstrate hyperproteinemia, hyperglycemia, or hyperlipidemia to suggest pseudohyponatremia. Interestingly, serum uric acid levels were normal. It was decided to begin treatment to differentiate between SIADH and CSWS with plans to initiate normal saline in the setting of worsening hyponatremia below 128 mEq/L.

**Figure 2:**
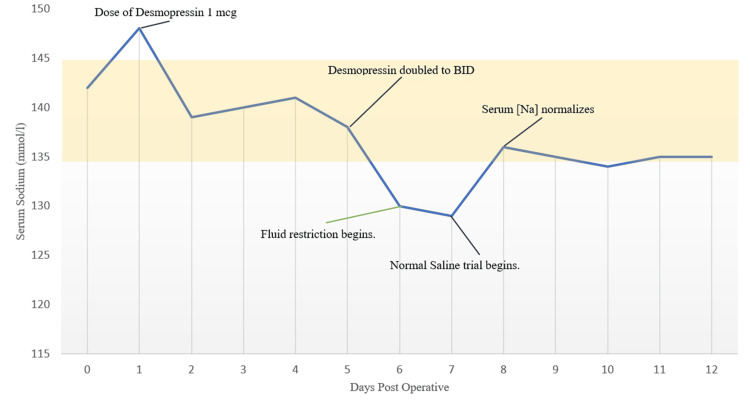
Serum sodium and response to desmopressin and fluid restriction and administration since the first pituitary adenoma resection BID: twice per day

Following the initiation of normal saline to differentiate this patient’s electrolyte status, her sodium began up trending back to normal limits, supporting the diagnosis of CSWS over SIADH, as outlined in Figure 2. Unexpected to her clinical diagnosis, her hyponatremia presented in the setting of isotonic hypovolemia, a deviation of typical CSWS that is characteristically hypotonic.

**Figure 3:**
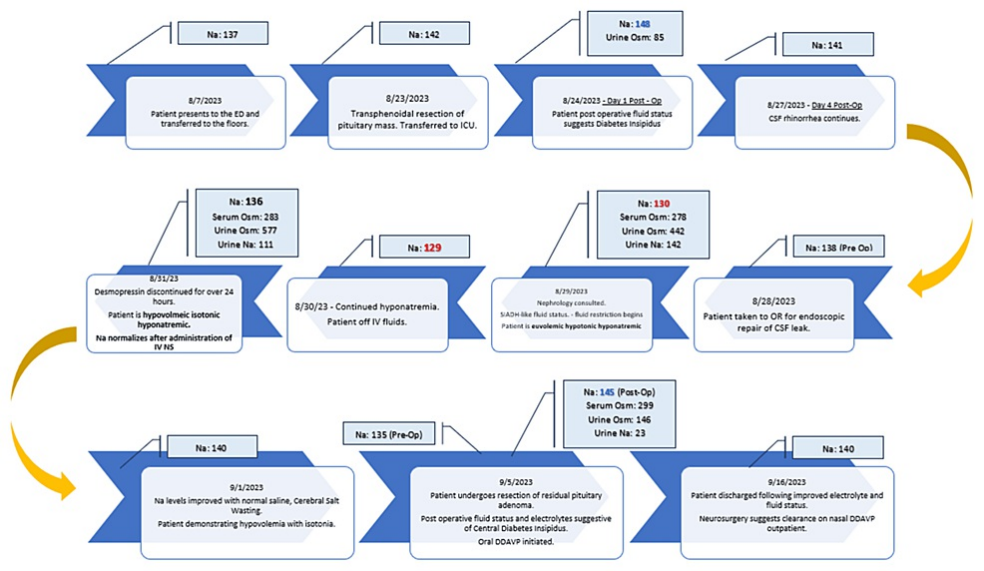
Serum sodium and response to desmopressin and fluid restriction and administration since the first pituitary adenoma resection Image credit: Gerik Cervantes ED, emergency department; ICU, intensive care unit; Osm, osmolality; CSF, cerebrospinal fluid; NS, normal saline; SIADH, syndrome of inappropriate anti-diuretic hormone

Later in her hospital course, an MRI of the brain revealed residual pituitary macroadenoma, a source of CSF leak and pneumocephalus (Figure 1D, 1E, 1F). Thus, reoperation to remove the residual tumor was subsequently performed. The recurrence of previously diagnosed central diabetes insipidus occurred postoperatively as the patient started experiencing symptoms of polydipsia, polyuria, and rising sodium levels. Sodium began increasing, and serum osmolality, urine osmolality, and urinary sodium were elevated as listed in Table 1. The patient was subsequently restarted on subcutaneous desmopressin. Sodium levels were normalized, and the patient was transitioned to oral desmopressin. Neurosurgery cleared the patient to use intranasal DDAVP, and the patient was transitioned from oral to intranasal DDAVP before being transferred out of the ICU. The clinical course is highlighted in Figure 3.

## Discussion

Hyponatremia is commonly seen in pathologies involving intracranial structures that often lead clinicians to diagnose SIADH in the setting of neurological disease. It is not uncommon for this syndrome to occur, potentially even expected when seeing patients with hyponatremia. Considering the similarities between SIADH and CSWS, it is crucial to isolate a proper diagnosis as delayed treatment can worsen hyponatremia and further lead to clinical deterioration including obtundation, seizures, coma, and respiratory arrest [7]. Both conditions are treated differently. Patients with SIADH are usually treated with fluid restriction and/or vasopressin receptor antagonists, while patients with CSWS should be treated with 3% saline to raise serum sodium and theoretically suppress the release of ADH [8]. Fluid restriction has been shown to worsen CSWS by increasing sodium excretion and can even cause hypotension [6]. Laboratory values present similarly in both conditions including low serum sodium, serum osmolarity less than 280 mOsm/kg, urine osmolarity greater than 100 mOsm/kg, and urine sodium greater than 40 mEq/L [9].

Hypovolemia and the restoration of euvolemia through a trial of isotonic saline administration can suggest CSWS over SIADH, which made CSWS the most likely diagnosis of our patient [5]. Furthermore, in the setting of isotonic hyponatremia, sometimes referred to as pseudohyponatremia, it is important to assess for conditions of hyperproteinemia and hyperlipidemia, which demonstrate hyponatremia in a state that serum sodium is actually normal. Both of these states were ruled out in the evaluation of this patient with lipid studies and urinalysis showing no abnormalities, suggesting hyperproteinemia or hyperlipidemia, prompting further assessment. During evaluation for the potential of a diagnosis of SIADH, serum uric acid concentration has a role in diagnostic utility. It has been reported in multiple studies that the value of serum uric acid remains low in SIADH, and in conditions of volume depletion, the contrary is seen with laboratory values consisting of hyperuricemia [5,10]. Laboratory values in the evaluation of our patient for serum uric acid level were within normal limits, further lessening the diagnosis of SIADH [11]. One limitation in our case is that we did not calculate the fraction excretion of urea (FEUrea) to support our diagnosis. In CSWS, you would expect FEUrea to remain elevated after the correction of hyponatremia in contrast with SIADH in which you expect the normalization of FEUrea. We did not pursue these laboratory values because the correction of hyponatremia through volume expansion was convincing enough to point toward CSWS diagnosis.

It is not uncommon to assess for SIADH following pituitary surgery, specifically a transsphenoidal resection of a mass. There have been multiple cases in the literature that discuss hyponatremia following pituitary surgery. The rarity in this presentation follows the stark progression of a hypernatremic state to hyponatremia. While DDAVP can cause hyponatremia in cases of overuse, the further down trending serum sodium, even after discontinuation, was unexpected and caught the critical care team, neurosurgery, and nephrology by surprise. The clinical team realized the worsening hyponatremia far extended the limits of DDAVP in half-life as well, further causing concerns of thorough investigation leading to our diagnosis. While CSWS is not an undocumented occurrence, our presentation highlights multiple areas of rarity. This unique and novel presentation of CSWS should be taken into consideration as a reminder to neurosurgery to extend further considerations in the evaluation of hyponatremia and hypernatremia, whose awareness of CSWS should be strongly investigated. Following a thorough literature review, cases that described and supported CSWS following pituitary surgery were all associated with a hypovolemic state supported with clinical assessment and laboratory values; however, none presented initially with hypernatremia [12-15]. This makes the discussion, regarding our patient, incredibly rare while also increasingly important and should be strongly advised to all involved in the evaluation of evolving serum sodium levels.

## Conclusions

When dealing with hyponatremia, caution should especially be advised in cases following neurological surgery. Taking into consideration CSWS as a potential complication is crucial, as the worsening of hyponatremia due to incorrect treatment has led to unfavorable outcomes. Given the basis that the treatment for CSWS is fundamentally different than SIADH, differentiation between these syndromes can prevent further mortalities. It is vital to understand both processes in managing patients following evaluation with serum osmolality, urine osmolality, and urine sodium in clinical scenarios that mimic this condition.
